# Intestinal Dysbiosis Disguised as a Rectal Fistula Treated With Autologous Fecal Microbiota Transplantation

**DOI:** 10.7759/cureus.14115

**Published:** 2021-03-25

**Authors:** Nicolina Scibelli, Pratishtha Singh, Kathleen Raynor

**Affiliations:** 1 Internal Medicine, Grand Strand Medical Center, Myrtle Beach, USA; 2 Gastroenterology, Grand Strand Medical Center, Myrtle Beach, USA

**Keywords:** intestinal dysbiosis, autologous fecal microbiota transplant, fecal microbiota transplant, fecal microbiota transplantation, fmt, sigmoid-end colostomy, intestinal microbiome, gut microbiome

## Abstract

Fecal microbiota transplantation (FMT) has been efficacious in the treatment of intestinal dysbiosis, derangement of the native intestinal microflora, and the indications for autologous FMT are growing. A 69-year-old Caucasian man with a past medical history of paraplegia secondary to motor vehicle accident and sigmoid-end colostomy presented to his gastroenterologist with the complaint of rectal discharge. A complicated medical course pre-dated his presentation and included multiple decubitus ulcers requiring debridement and several courses of broad-spectrum antibiotics. The rectal discharge was initially presumed to be from a fistula leading to one of his ulcers; however, workup with anoscopy, flexible sigmoidoscopy, and magnetic resonance imaging of the pelvis showed no visible perirectal abscess or connection to the sigmoid colon through a fistula. Intestinal dysbiosis was an alternative theory considered to be the cause of his copious rectal discharge due to his several courses of broad-spectrum antibiotics and prolonged inactivity of his gut. This prompted a trial treatment plan utilizing autologous FMT, with the patient administering enemas containing his own stool to the distal limb of his bowel. As a result of this treatment, the patient’s chief complaint completely resolved within days of initiating treatment, although symptoms did eventually return. We would like to propose that further randomized studies should be done to investigate autologous FMT as a treatment for patients suffering from intestinal dysbiosis following sigmoid-end colostomy.

## Introduction

Currently, the most common indication for a fecal microbiota transplantation (FMT) is recurrent *Clostridium difficile* infection [1]. FMT is typically performed by infusing donor stool into the recipient’s gastrointestinal tract via a colonoscopy, although it can also be performed through an upper gastrointestinal (GI) approach [2]. These patients suffer from a disruption in their intestinal microflora due to recurrent use of antibiotics, also referred to as intestinal dysbiosis. The introduction of heterogeneous bacteria from FMT has been shown to re-colonize the GI tract with an ideal microbiome, thus preventing *C. difficile* overgrowth [1]. As a result of the success seen with FMT in the treatment of *C. difficile*, its utility in treating intestinal dysbiosis has expanded. FMT has been applied in various extra-intestinal conditions, including metabolic diseases, neuropsychiatric disorders, autoimmune diseases, allergic disorders, Parkinson’s disease, multiple sclerosis, myoclonus dystonia, chronic fatigue syndrome, idiopathic thrombocytopenic purpura, and tumors [3]. Autologous FMT is a newer application being used in the treatment of patients undergoing hematopoietic stem cell transplantation (allo-HSCT) and has had some consideration in patients with inflammatory bowel disease (IBD) [4,5]. This method uses one’s own feces to help restore the microbiome of the gut. We present this case to consider further application of autologous FMT in cases of rectal discharge after end-colostomy placement.

## Case presentation

A 69-year-old Caucasian male presented to his outpatient gastroenterology office with complaints of copious rectal discharge and for discussion of treatment options. His medical history was significant for trauma leading to a T7 spinal cord injury resulting in a thoracic arteriovenous malformation requiring surgical intervention. His postoperative course was complicated by paraplegia leading to him being constricted to a wheelchair. Twenty-five years after his initial surgery, the patient developed a left ischial ulcer and a sacral decubitus ulcer with concomitant methicillin-sensitive *Staphylococcus aureus* (MSSA) bacteremia, which was treated surgically with multiple debridements and prolonged courses of broad-spectrum antibiotics. During this time, the patient also underwent sigmoid end-colostomy placement due to chronic constipation. After another three years without complications, he presented with a new ischial wound and fevers, with wound cultures positive for MSSA, *Streptococcus agalactiae*, and *Morganella morganii*. He was treated with incision and drainage along with another eight-week course of ceftriaxone and metronidazole. In the following months, he began to have new onset of copious, purulent rectal discharge, leading to poor healing and further debridement procedures for his wounds.

When inquiring further, the patient previously reported minor mucus drainage from the anus, occurring about once every three weeks. However, in the months leading to his presentation, his purulent fluid drainage increased in both volume and odor, with up to estimated 1 L daily of purulent rectal discharge. He was referred to a colorectal surgeon due to above-mentioned complaints. On presentation, his physical examination was notable for paraplegia, flexion contracture of bilateral knees, and a well-pouched colostomy in the left lower quadrant of the abdomen. No parastomal hernia was noted. His rectal examination was notable for a normal-appearing anus with some minor residual hemorrhoidal skin tags and obvious atrophy of both buttocks. Digital rectal exam (DRE) demonstrated very low rectal tone without sensation and a large amount of thin, yellow purulent drainage from the rectum. A right ischial ulcer was present, measuring 5 cm in diameter, tracking medially and superiorly with easily palpated underlying bone.

**Figure 1:**
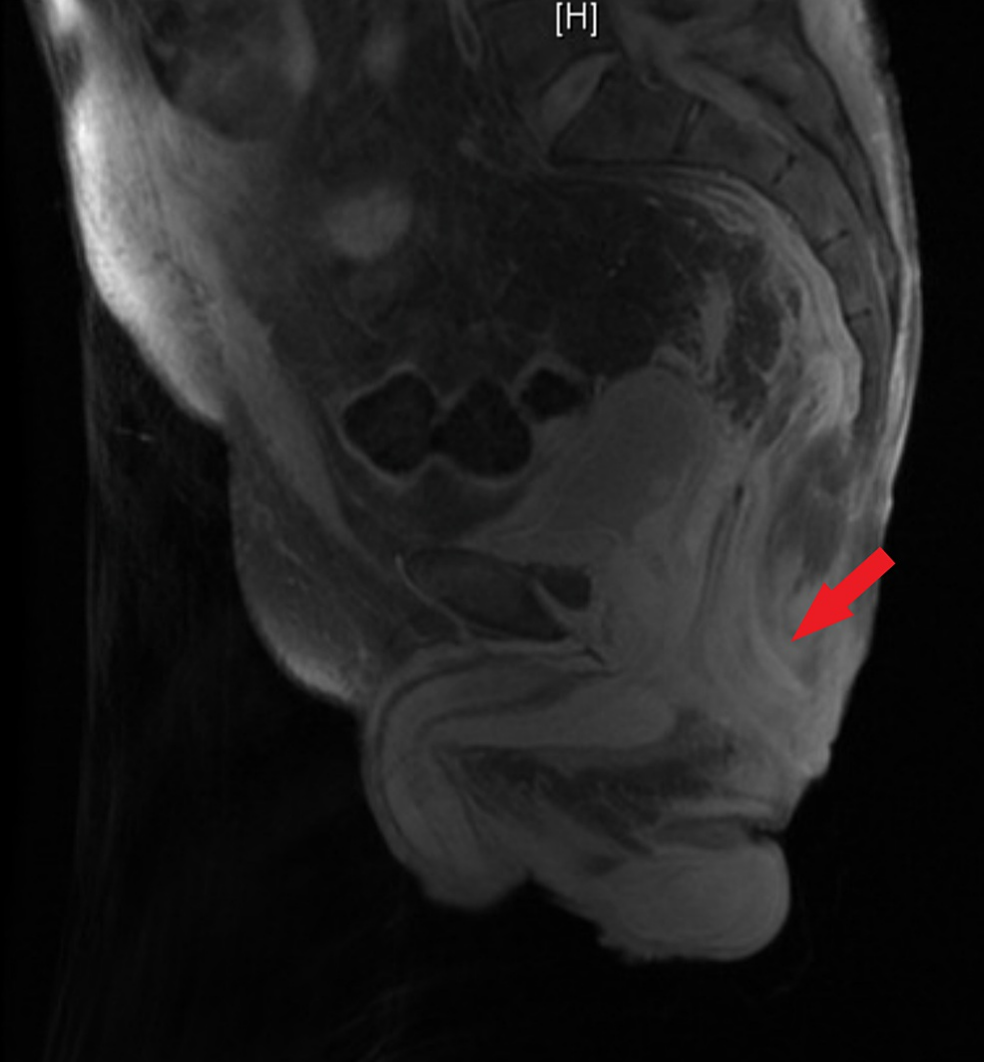
Sagittal view of pelvic MRI. Red arrow indicates the rectal stump without any visible abscess or fistula. MRI, magnetic resonance imaging

**Figure 2:**
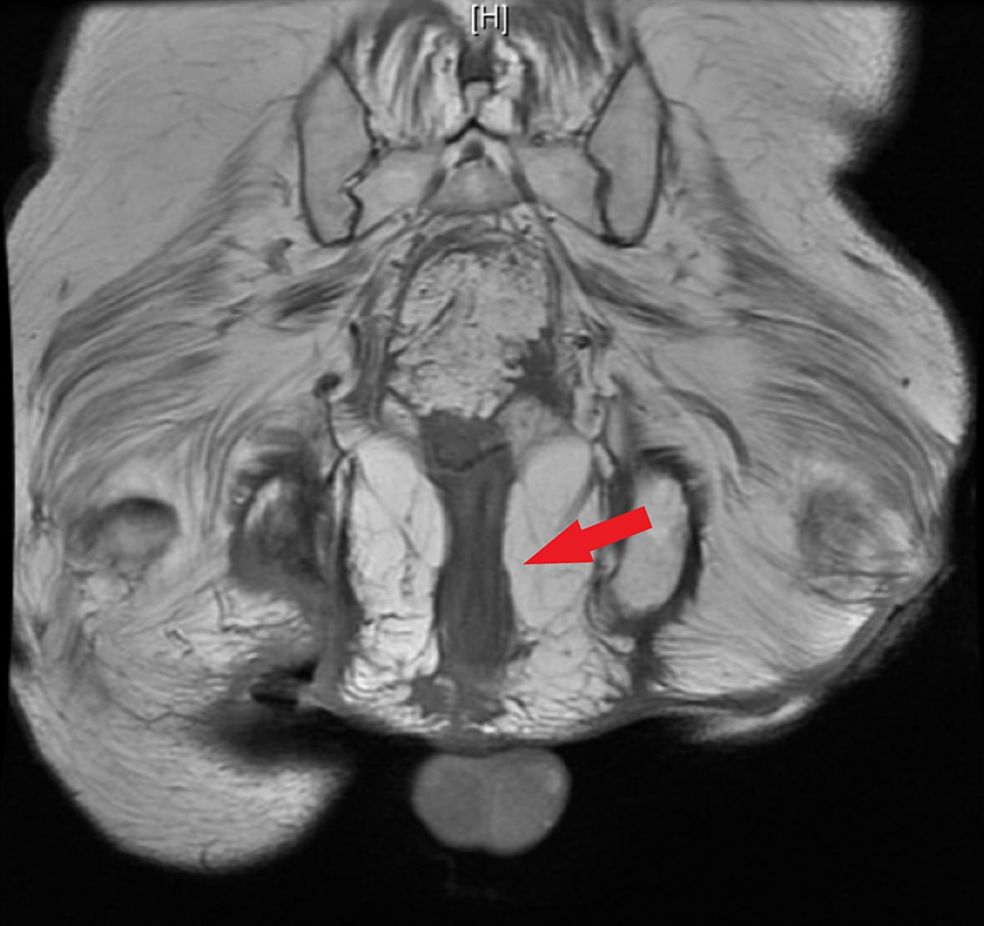
Coronal view of pelvic MRI. Red arrow indicates the rectal stump without any visible abscess or fistula. MRI, magnetic resonance imaging

An anoscopy was performed revealing a large amount of purulence from the rectal wall in the right posterior location. Due to this, there was a concern for a rectal abscess with fistula and a magnetic resonance imaging (MRI) of the pelvis without contrast was obtained which revealed a soft tissue ulceration in the right ischial region extending from the skin to the ischial tuberosity and no evidence of focal fluid collection to suggest drainable abscess or other fluid collection (Figures 1, 2). The patient underwent further evaluation with an anorectal examination under anesthesia and a flexible sigmoidoscopy (FS). DRE produced a large amount of purulent drainage, FS confirmed the same purulent fluid to the top of the rectal stump, and the fluid was sent for gram stain and culture. No connection to the sigmoid colon was found, thus confirming that the colostomy was in fact an end colostomy. The rectal mucosa appeared atrophied and with some diversion proctitis, but no evidence of granulation tissue or pseudomembranes, and no clear source for the purulence was discovered. The healed abscess cavity of the right ischial decubitus ulcer was aspirated and did not show evidence of purulent fluid. After a thorough examination as noted above and an X-ray barium enema, it was determined that there were no clear fistulous connections/fistula, and the working diagnosis included infectious versus inflammatory proctitis. His fluid drainage was positive for extended-spectrum beta lactamase (ESBL) *Citrobacter freundii* and *Proteus mirabilis* requiring a four-week course of ertapenem.

At this point, it was suspected that intestinal dysbiosis may be contributing to the patient’s current symptoms and a decision was made to treat him with autologous FMT. This involved daily enemas with a mixture of approximately 50 cc of feces from his ostomy with 50 cc of distilled water and retained for 20 minutes or as long as possible. The patient noted immediate improvement within two days when the rectal discharge had stopped completely. After approximately two weeks, patient began to have reoccurrence of his rectal discharge and the results of the enemas proved not to be permanent. The patient and his colorectal surgeon ultimately decided to move forward with a rectal mucosectomy, removing the mucosa of the rectum. The patient had slow wound healing following this procedure but decreased rectal discharge.

## Discussion

Intestinal dysbiosis is typically defined as alterations of the gut microbiota that can occur secondary to diet, toxins, drugs, and pathogens. These exposures can then result in local and systemic inflammation, further disturbing the barrier function of the gut. Enteric pathogens have been noted to be the prevailing cause of dysbiosis in animal models [6]. Antibiotics can also contribute to the disruption of the microbiome both directly as well as indirectly and contribute to the overpopulation of such enteric pathogens. Directly, microbial diversity can be depleted due to the broad-spectrum activity of antibiotics. Both pathogenic bacteria and commensal bacteria are indiscriminately killed. Indirectly, the homeostatic state of the gut can be disturbed and bacteria that once lived through symbiosis and codependency may no longer have the necessary nutrients they require to survive. Through these mechanisms, commensal bacteria and sensitive strains of pathogenic bacteria are eliminated, while antibiotic-resistant strains of bacteria will have a growth advantage in this unstable microbiome [7]. In our patient, it can be assumed that after multiple rounds of empiric antibiotics, deleterious effects to the microbiome developed. Broad-spectrum antibiotics were necessary due to his decubitus ulcers with osteomyelitis, but in doing so, his bacterial flora was decimated. This disruption led to the emergence of pathogenic bacteria that were resistant to the empiric antibiotics being given. ESBL *C. freundii* and *P. mirabalis* were cultured, requiring ertapenem for treatment as they were likely contributing to our patient’s dysbiosis.

Dysbiosis is also seen after prolonged inactivity of the gut. Studies performed on patients who underwent loop ileostomy showed that the de-functioned intestine had significant atrophy compared to functional tissue. Bacterial load was reduced by 62.4%, and microbial diversity was also decreased in the inactive bowel, likely from prolonged starvation [8]. This is a similar phenomenon to what occurs to the bacterial biodiversity of the gut when patients are fed with total parenteral nutrition (TPN). Profound dysbiosis was noted for an average of one year in patients who received TPN for greater than two months [9]. Our patient had very clear intestinal atrophy in the distal, or efferent, bowel limb as visualized during FS. Also clearly seen during that procedure was a blind pouch with no connection to the proximal portion of the gut, confirming end colostomy. This validated that there were no nutrients provided to the distal arm of the gut, thereby leading to starvation and large decrease in both bacterial load and bacterial diversity.

In a controlled study, autologous FMT was used in patients undergoing allo-HSCT treatment to treat dysbiosis. Following treatment with allo-HSCT, gut microbiota diversity was severely depleted due to routine infectious prophylaxis with antibiotics from immunosuppression or empiric treatment with clinical reasoning. Prior to the allo-HSCT treatment, the patient’s own feces was collected. Autologous FMT was performed with the feces and the percentage of recovery of the patient’s original microbiome was compared to recovery of patients who did not receive the intervention. The control arm that did not receive autologous FMT saw only 27% of patients regain a bacterial composition 75% or more similar to their initial fecal sample, whereas 79% of the autologous FMT treated patients had a 75% or more recovery [5]. In IBD, studies have taken a patient’s own stool during a time when the patient is in remission and then re-introduced to the patient when they are having a flare. In comparing autologous to heterologous FMT, which has previously shown cure rates as high as 90.9%, autologous FMT has cure rates as high as 90% in some patient cohorts. In addition, the transmission of communicable and noncommunicable diseases is avoided with the use of autologous FMT [4].

## Conclusions

Our patient, who saw a great benefit from autologous FMT, served as an ideal patient. We theorize that an overgrowth of pathogenic bacteria after broad-spectrum antibiotic use occurred in the distal or efferent bowel limb. This inactive portion of bowel was especially susceptible to dysbiosis as it was deprived of enteral nutrition. As seen during the FS, the mucosa was atrophied, consistent with a starved bowel that had a large decrease in both bacterial load and diversity. By performing enemas composed of his own feces, symptoms improved temporarily and the patient experienced improved quality of life. Although for this patient the solution was not permanent, it was low risk compared to surgical intervention. We propose that further randomized studies should be done to investigate the utility of autologous FMT in patients suffering from intestinal dysbiosis after end colostomy in the distal limb of the intestine, especially if initiated early.
